# Transient ischaemic attack in a patient with known temporal arteritis: a case report

**DOI:** 10.4076/1757-1626-2-8542

**Published:** 2009-08-20

**Authors:** Umesh T Kadam

**Affiliations:** Arthritis Research Campaign National Primary Care Centre, Keele UniversityStaffordshire ST5 5BGUK

## Abstract

**Introduction:**

Older populations are more at risk of problems such as temporal arteritis or polymyalgia rheumatica, and these conditions are often first diagnosed in general practice, with usual ongoing care and long-term treatment with oral steroids. These inflammatory conditions are also potential risk factors for other complicating presentations such as transient ischaemic attacks, but the precise comorbid links and how these might influence clinical management in general practice are unclear.

**Case presentation:**

An 82-year-Caucasian woman living alone requested a home visit after a single episode of speech disturbance and disorientation which lasted 15 minutes. This occurred 2 weeks after cessation of her oral prednisolone for temporal arteritis, which was clinically diagnosed in 2006 and later confirmed by a biopsy. She also had a past medical history of ischaemic heart disease based on symptom presentation and an abnormal ECG in October 2005, but no other relevant risk factors for cardiovascular disease.

**Conclusions:**

Transient ischaemic attack is an alternative presentation or complication of an inflammatory disease such as temporal arteritis. The clinical implications of this case relate to the assessment of comorbid risk in TA and in tailoring the drug treatment. In using prednisolone treatments in such patients, general practitioners will need to carefully titrate drug doses and the duration of treatment to prevent complications. Clear evidence for the precise type of management remains to be established.

## Introduction

Temporal arteritis (TA) and polymyalgia rheumatica are inter-related inflammatory disorders, which are managed over long term in general practice. The highest rates for new onset diagnosis for these conditions are in the age group 70 years and over, and in the general practice population are estimated at 6 per 10,000 person consulting years for temporal arteritis and 22 per 10,000 person consulting years for polymyalgia rheumatica, with a female to male ratio of around 2 to 1 [1]. The diagnosis of each is based primarily on clinical features at presentation, and supported additionally by erythrocyte sedimentation rate (ESR), thrombocytosis and biopsy of temporal artery [2,3]. The recommended treatment for both conditions is long-term oral prednisolone [4].

In temporal arteritis, immediate and high dose (60 mg daily) treatment is recommended for a month in a person with suspected TA to prevent the high risk of ocular complications [5], which can be tapered down gradually after the first month. The duration of therapy can last many years, but it is unclear as to how this condition may relapse according to the dose of prednisolone. There have been specific and isolated reports of other comorbid complications, which include cerebrovascular accidents [6] and cardiovascular events [7]. Despite these known complications, in general practice where most of these patients are managed over longer term, current approaches to drug dose management and prevention of complications seem empirical.

## Case presentation

A support carer requested a home visit on behalf of her client, who is a-82-year Caucasian lady living alone. Three days previously, in the presence of the support carer, she had had acute symptoms last 15 minutes during which she was alert but disorientated, could not express her speech, and afterwards found that writing had become difficult. There was no loss of consciousness and neither was there any weakness or paraesthesia in any of her limbs. She improved over a few hours, and on the day of the visit, she felt as if she was back to normal. She diagnosed herself as having had a transient ischaemic attack.

On closer questioning, she explained that for several years she had been on prednisolone for her eye problems (temporal arteritis) and 2 weeks previously this drug had been stopped after a period of reduction in the dose under GP supervision. She had been keen to come off them as she had suffered from dyspeptic symptoms.

On the day of the visit, her clinical examination was unremarkable, with the exception of a blood pressure measurement of 160/80. However her home BP recordings were in normal range. She is right handed, was alert and orientated, and her specific cardiac (e.g. sinus rhythm, no murmurs or carotid bruits) and neurological system (e.g. speech, power, writing) examinations were normal. There was no temporal artery tenderness.

Past medical history from her clinical records showed that she had been diagnosed in October 2005 with ischaemic heart disease (IHD) on the basis of atypical chest symptoms and an abnormal ECG showing inferior ischaemic changes. Her initial treatment for this was nitrolingual spray and aspirin, but she had refused a statin. In November 2006, she presented with visual symptoms and pain in her jaw and an ESR of 36, and was commenced on oral steroid with a provisional diagnosis of temporal arteritis. She responded well with resolution of her symptoms to this initial treatment, but in early 2007 wanted to reduce her dose of prednisolone because of dyspeptic symptoms. However some of symptoms returned and she was referred to the ophthalmology service for further confirmation of the diagnosis. Her temporal artery biopsy result came back as showing ‘inflammatory changes’ and together with her symptoms including the partial loss of vision in her left eye; she was given a formal diagnosis of TA and recommended to continue the prednisolone. To this treatment, she was given a proton inhibitor, and by now she had stopped using her nitrolingual spray.

She was married but had no children, never smoked and always been tea-total. She retired as a book-keeper many years ago and has carer support because of mobility problems, but she was ambulant around her house.

Based on her clinical history a diagnosis of transient ischaemic attack secondary to withdrawl of treatment for temporal arteritis was made, and with her agreement prednisolone 30 mg daily was re-commenced. Follow up at 1 week was arranged to consider referral for TIA clinic depending on her symptoms and blood results. During the home visit, routine blood samples were collected, which were later reported as an ESR of 27 and random cholesterol of 5.4.

Her clinical records were reviewed 2 weeks after the first visit, and showed that she had had no further symptoms, a referral to TIA clinic and simvastatin treatment had been offered, but both options were refused. Her prednisolone dose was now down to a maintenance dose of 7.5 mg daily.

## Discussion

This case illustrates the interface between the management approaches to single diseases and how comorbidity in the older person may weave complex relationships that influence the management as clinicians in primary care or otherwise.

Despite her lack of risk factors for cardiovascular disease, her first significant diagnosis was based on atypical symptoms and ECG signs. Whilst it is possible that this was a mis-diagnosis, the question in view of the later diagnosis of TA, is whether these symptoms heralded the underlying development of TA. There have been case reports in literature, which support the link in symptoms of angina and underlying arteritis [8].

She then presented with symptoms more consistent with TA but with a marginal increase in ESR, and then notably there was good therapeutic response to treatment with oral prednisolone. It was the clinical suspicion (specifically jaw pain) and good response to the drug intervention that initially supported the diagnosis of TA, despite the marginally raised ESR. In literature and guidelines, an ESR of over 50 and lymphocytosis, have been recommended as supporting a diagnosis of TA [2,3]. However, this case highlights that clinical skills and therapeutic response are as important as laboratory tests in the decision-making process, when gold standard investigation such as biopsy in TA is not feasible in all patients.

The clinical features that required the home visit were classical presentation of a transient neurological deficit, in the context of withdrawal of oral prednisolone. Here the challenge to clinical decision-making in a person of this age was on whether this was an ischaemic event with atherosclerotic basis or an event with inflammatory TA basis. She had a record of possible IHD and yet had no relevant risk factors with a return report of a marginally raised random cholesterol levels. So an immediate response would have been to arrange a TIA clinic assessment that focused on the management of atherosclerosis pathway or instead the alternative management of the acute event with the use of oral prednisolone. The decision made at home visit was to treat as if there was a secondary TA event and to follow-up with results and symptom re-assessment.

The other key issues raised are the dose and duration of oral prednisolone therapy in patients with TA, in both in the initial and continuing phases. Current literature recommends high dose (60 mg daily) for one month in the new event of TA, but in this case had had recent treatment lasting two-and-half years and she also had a history of possible side effects, resulting in the decision to prescribe 30 mg prednisolone daily [3]. Current consensus also advises that the course of TA can last months or years and that there should be a gradual tapering down of the dose [9]. Yet, it is unclear as to what the minimum dose of prednisolone might be indicated for patients with comorbidity, for example diabetes, or those who suffer associated side effects. Furthermore, how the dose of prednisolone can be tailored over time and how the flare-ups of TA disease are monitored, remains to be established. The crucial learning point in this case is arguably that in the single pursuit of TA and decreasing therapy, this patient may have suffered an associated risk and complicating comorbidity of an event such as transient ischaemic or neurological deficit.

Finally, developments in science and the rawness of information in general practice encounters have to be placed in the context of the patient decision, which is final. So this patient had her own views on the choice and dose of therapy, on whether she was referred or not, and whether she consented to the guidelines that clinicians are recommended to follow.

## Conclusions

Transient ischaemic attack is an alternative presentation or complication of an inflammatory disease such as temporal arteritis, and there have been case reports of cardiac presentations of TA. The clinical implications of this case relate to the assessment of comorbid risk in TA and in tailoring the drug treatment. In using prednisolone treatment in such patients, general practitioners will need to carefully titrate drug doses and the duration of treatment to prevent such complications, but clear evidence for the precise type of management remains to be established.

## Abbreviations

ESR: erythrocyte sedimentation rate
IHD: ischaemic heart disease
TA: temporal arteritis
TIA: transient ischaemic attack
